# Bi-directional freezing affords elastic and durable artificial tooth enamel

**DOI:** 10.1038/s42004-022-00663-x

**Published:** 2022-04-04

**Authors:** Teresa S. Ortner

**Affiliations:** Communications Chemistry, https://www.nature.com/commschem

## Abstract

Tooth enamel possesses outstanding viscoelasticity as well as stiffness — a combination of properties often considered a trade-off. Now, a bi-directional freezing method affords a biomimetic composite with mechanical properties superior to those of enamel.

Researchers have attempted to create materials with stiffness, hardness, and viscoelasticity akin to tooth enamel by mimicking the parallel arrangement of nanorods in the natural material^1,2^. The utility of the obtained composites, however, has been limited by their sub-millimeter thicknesses. To make a material macroscopically as stable as enamel, while also being machinable, remains an enormous challenge. Now, Lin Guo, Xuliang Deng, and colleagues from Beihang University and Beijing Laboratory of Biomedical Materials in China, together with Nicholas Kotov from the University of Michigan, USA report a bulk composite material that is stronger, harder, and more durable than enamel (10.1126/science.abj3343)^3^.

“Enamel gets its mechanical property combination from its hierarchical structure: parallel hydroxyapatite nanowires interconnected by confined biomolecules, a so-called amorphous intergranular phase,” explains Guo. “The nanorods forming the enamel are aligned parallel to each other in the bulk phase, which is hard to replicate in synthetic mimics,” says Kotov. Now, the team exploits a bi-directional freezing method^4^, where a polydimethylsiloxane wedge substrate induces a temperature gradient in a polyvinyl alcohol dispersion of hydroxyapatite nanowires. This bi-directional temperature gradient directs the ice crystal growth, which in turn forces the nanowires to occupy the gaps between ice-lamellae in a parallel fashion as depicted in Fig. 1. The hydroxyapatite nanowire scaffold is chemically modified by coating the strands with amorphous zirconia prior to their arrangement. “Controlled growth of the amorphous layer on the surface of the nanorods was an essential tool to engineer the interfaces that are a replication of those in tooth enamel and enable efficient dissipation of vibrations,” says Kotov.

**Fig. 1:**
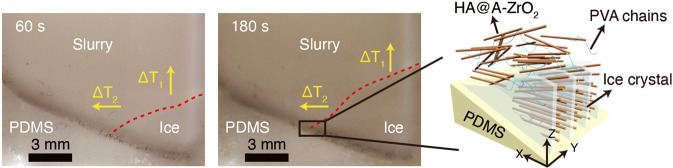
Assembly of the nanorod structure. A temperature gradient is induced by a PDMS wedge-shaped surface, which causes a temperature gradient within the nanorod-dispersion on top. Solvent ice crystals form in parallel lamellae when the temperature sinks. The hydroxyapatite nanorods coated with an amorphous layer of zirconium dioxide (HA@A-ZrO2) assemble in between the ice lamellae.

The composite has a Young’s modulus of 105.6 ± 12.1 GPa, a hardness of 5.9 ± 0.6 GPa, a flexural strength of ~142.9 MPa, and a fracture strain of 0.018. All of these values exceed tooth enamel. “Engineering this machinable composite from the nano to the macroscale is a critical step towards its technological realization, for example for biomedical implants,” says Kotov. Indeed, the bi-directional freezing method is fast and has the potential for mass production.

“Tooth enamel not only possesses a hierarchical structure, but it also has a chemical gradient throughout the outer and inner shells,” says Guo. The team now aims to mimic tooth enamel even more closely by incorporating constituents of this gradient, such as Mg^2+^, which was recently found to play a key role for enamel’s mechanical properties, into the amorphous intergranular phase^5^.
